# Prognostic role of lncRNA TUG1 for cancer outcome: Evidence from 840 cancer patients

**DOI:** 10.18632/oncotarget.17844

**Published:** 2017-05-13

**Authors:** Jia Liu, Jieru Lin, Yingqi Li, Yunyuan Zhang, Xian Chen

**Affiliations:** ^1^ Department of Pharmacy, Guiyang Maternal and Child Health Care Hospital, Guiyang 550003, China; ^2^ Department of Respiratory and Critical Care Medicine, Guizhou Provincial People's Hospital, Guiyang 550002, China; ^3^ Department of Clinical Laboratory, The Affiliated Hospital of Qingdao University, Qingdao 266003, China; ^4^ Department of Ophthalmology, The Affiliated Hospital of Guizhou Medical University, Guiyang 550004, China

**Keywords:** cancer, prognosis, long non-coding RNA, taurine upregulated gene 1

## Abstract

LncRNA TUG1 has been demonstrated to be aberrantly expressed in several types of cancer and maybe serve as a prognostic marker for cancer patients. However, most individual studies have been limited by small sample sizes and controversial results. Therefore, this meta analysis was conducted to analyze available data to delineate the potential clinical application of lncRNA TUG1 on cancer prognosis, lymph node metastasis and tumor progression. Up to February 20, 2017, literature collections were conducted by comprehensive searching electronic databases, including Cochrane Library, PubMed, Embase, BioMed Central, Springer, ScienceDirect, ISI Web of Knowledge, together with three Chinese databases. The hazard ratios (HR) with 95% confidence interval (95% CI) were calculated to assess the strength of the association. Eight studies with a total of 840 cancer patients were included in the present meta analysis. The results indicated that elevated lncRNA TUG1 significantly predicted unfavorable overall survival (OS) (HR = 2.06, 95% CI: 1.23–3.45, *P* = 0.006), but failed to show incline to lymph node metastasis (HR: 1.16, 95% CI: 0.82–1.62, *P* = 0.40) and disease progression (III/IV vs. I/II: HR 1.16, 95% CI: 0.74–1.81, *P* = 0.52). In stratified analyses, a significantly unfavorable OS associated with elevated lncRNA TUG1 was observed in both bladder cancer (HR = 2.98, 95% CI: 1.84–4.83, *P* < 0.0001) and other system cancer (HR = 2.63, 95% CI: 1.42–4.87, *P* = 0.002), but not respiratory system cancer (HR = 0.93, 95% CI: 0.30–2.82, *P* = 0.895). The results indicated that increased lncRNA TUG1 was an independent prognostic biomarker for unfavorable OS but may not susceptible to lymph node metastasis and tumor progression in cancer patients.

## INTRODUCTION

Until now, cancer is still a major problem for public health all over the world, due to the increasing mobility and mortality [1]. In 2017, 1,688,780 new cancer cases and 600,920 cancer deaths are projected to occur in the United States [2]. The long term survival rate remains low in various types of cancer, and numerous scientists are dedicated to searching new potential biomarkers for early diagnosis and accurate prognosis prediction for cancer patients [3, 4].

Long noncoding RNAs (lncRNAs), which constitute the majority of transcripts encoded by the human genome, are non-protein coding RNA molecules greater than 200 nucleotides in length [5]. With the rapid development of genome-wide analysis technology, exponential growth of studies have been presented to suggest that lncRNAs are important regulatory molecules at every level of cellular physiology, including alternative splicing, cell cycle control, chromatin modification, dosage compensation, gene imprinting, genome rearrangement, and nuclear-cytoplasmic trafficking [6–8]. Moreover, lncRNAs have sparked considerable attention as vital modulators in carcinomas due to the potential role of lncRNAs in tumor development, progression, and metastasis [9–11].

Increasing clinical studies indicate that elevated expression of lncRNA TUG1 is closely linked with poor prognosis and high risk of cancer metastasis in many types of carcinomas [12]. However, most individual studies assessing the implication of lncRNA TUG1 levels in cancer have been limited by small sample sizes and controversial results. To our knowledge, no systematic meta-analysis has been conducted to evaluating the relationship between lncRNA TUG1 and the relevant clinical outcomes. Accordingly to this, it is necessary to perform a meta analysis to elucidate the clinical feasibility of lncRNA TUG1 as a putative biomarker candidate by systematically summarizing all eligible articles.

## RESULTS

### Included literatures

**Figure 1:**
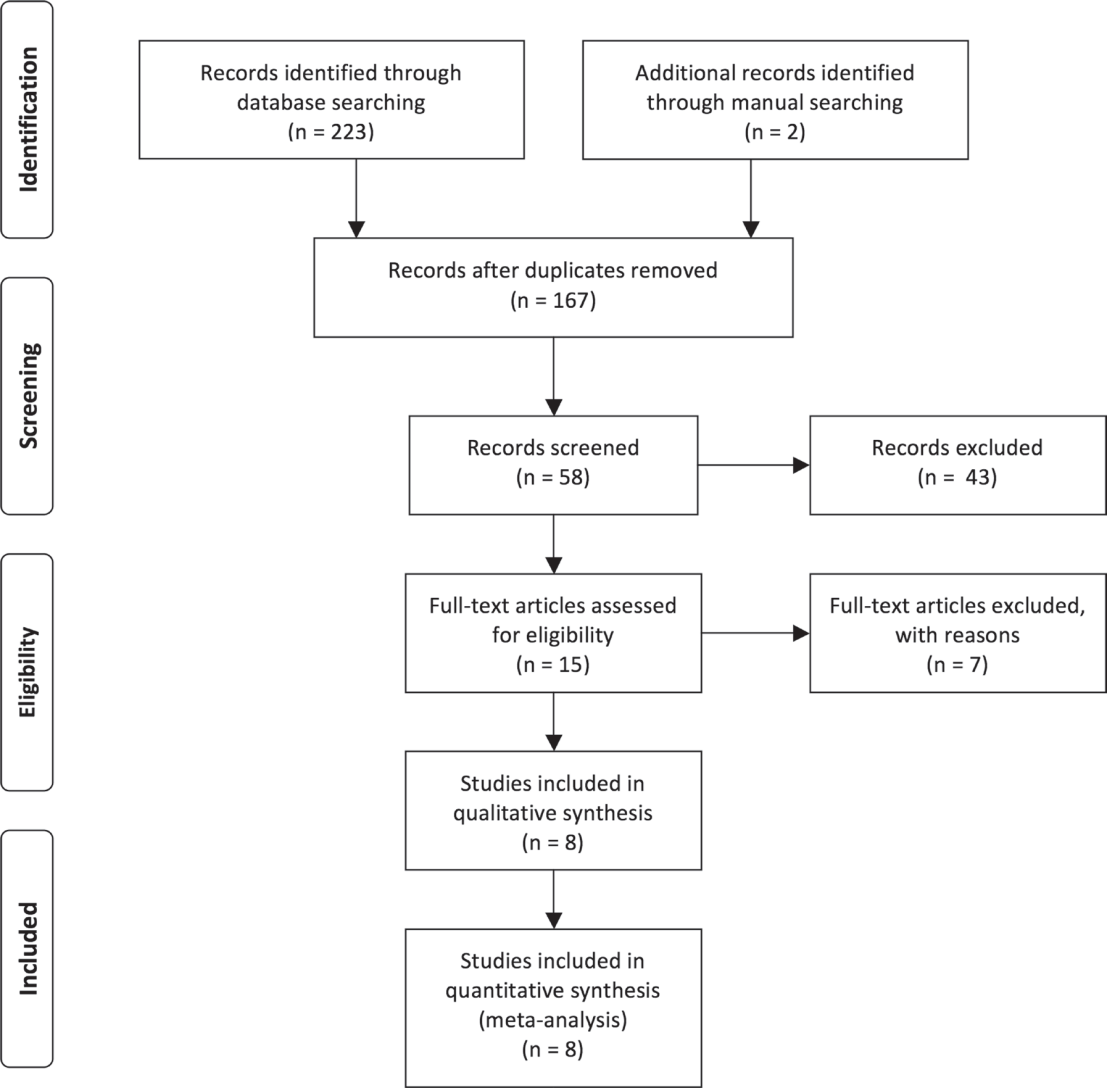
Flow diagram of the study search and selection process

Figure 1 presented the literature screening and study selection process. The initial search from electronic databases retrieved a total of 225 studies concerning the prognosis or metastasis of lncRNA TUG1 and cancer. After carefully screening the titles and abstracts, 210 articles were excluded because they were basic studies, letters, duplicate articles, reviews, or irrelevant to the present study. Full texts of the remaining 15 articles were further reviewed and assessed, and 7 articles were then removed because lncRNA TUG1 was not a dichotomic variable in the original studies. Ultimately, 8 articles were included in the current analysis [13–20].

### Characteristics of the enrolled studies

The main characteristics of eligible articles were summarized in Table 1. In summary, the sample sizes of these articles ranging from 33 to 218. All of the 840 patients were divided into high or low lncRNA TUG1 group according to the lncRNA TUG1 measurement results. Seven of eight studies were from China and related to 7 kinds of carcinomas, including osteosarcoma, gastric cancer, bladder cancer, colorectal cancer, small cell lung cancer, non small cell lung cancer and esophageal squamous cell carcinoma. Notably, the cut-off values were different, with median was applied in most articles.

**Table 1:** Summary of the eight included studies

Study	Origin of population	Study design	Disease	*N*	Stage	TUG1 assay	Survival analysis	Metastasis analysis	Hazard ratios	Follow-up Months
Zhang 2014 [13]	China	R	NSCLC	192	I/II, III/IV	qRT-PCR	OS	LNM	K-M	60
Tan 2015 [14]	China	R	BC	54	I–IV	qRT-PCR	OS	NA	K-M	60
Zhang 2016 [19]	China	R	GC	100	I/II, III/IV	qRT-PCR	OS	LNM/DM	K-M/HR	60
Sun 2016 [18]	China	R	CC	120	NA	qRT-PCR	OS	LNM	K-M	60
Ma 2016 [17]	China	R	OSA	76	I/II, III/IV	qRT-PCR	OS, PFS	IM	K-M/HR	60
Jiang 2016 [15]	China	R	ESCC	218	I/II, III/IV	qRT-PCR	OS	LNM	K-M/HR	80
Iliev 2016 [16]	Czech Republic	R	BC	47	I/II, III/IV	qRT-PCR	OS	DM	K-M	150
Niu 2017 [20]	China	R	SCLC	33	NA	qRT-PCR	OS	NA	K-M	30

Study design is described as retrospective (R); OSA, osteosarcoma; GC, gastric cancer; BC, bladder cancer; CC, colorectal cancer; SCLC, small cell lung cancer; NSCLC, Non small cell lung cancer; ESCC, esophageal squamous cell carcinoma; DM, distant Metastasis; LNM, Lymph Node Metastasis; IM, Initial metastasis

### Meta analysis results

**Figure 2:**
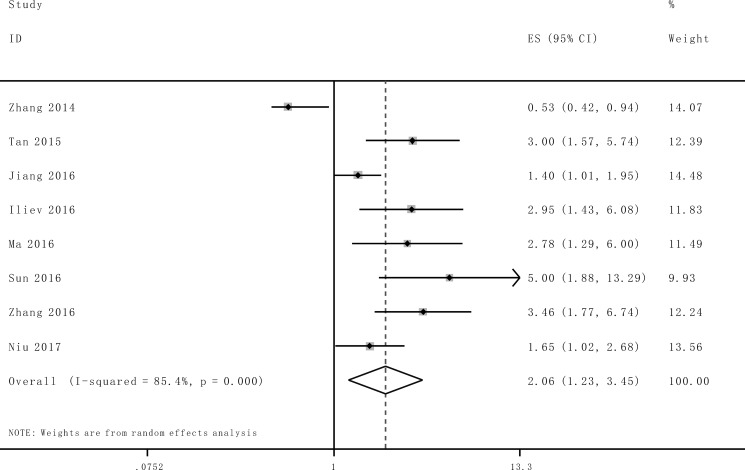
Forest plot for the association between TUG1 expression with overall survival (OS)

As indicated in Figure 2, *I*^2^ values for OS was 85.4%. Therefore, a random effects model was employed to analysis the pooled HR and its 95% CI due to the existence of significant heterogeneity among those 8 studies which involved in OS analysis. Enforced lncRNA TUG1 expression was predictive of unfavorable OS in various carcinomas with multivariate analysis (HR = 2.06, 95% CI: 1.23–3.45, *P* = 0.006).

**Figure 3:**
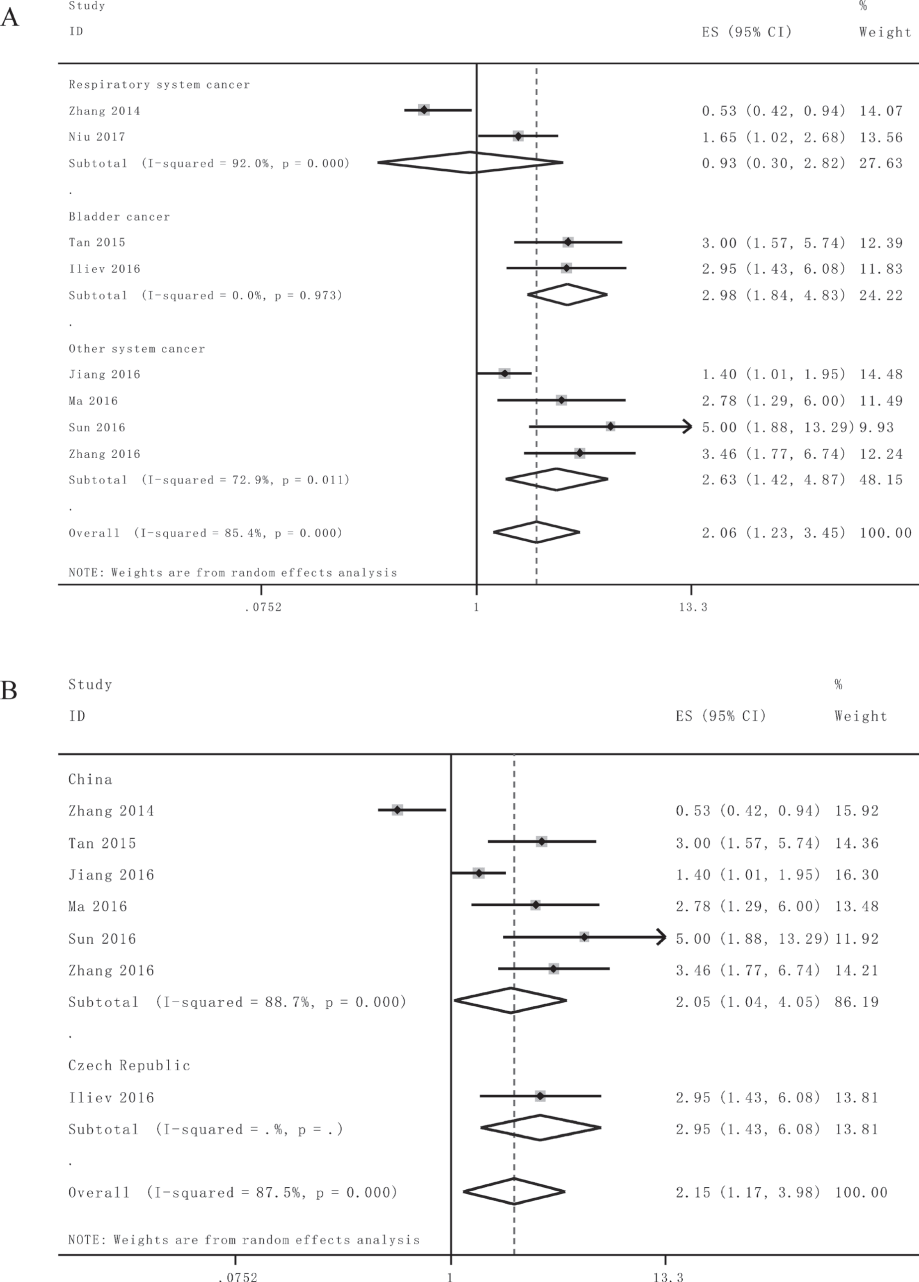
Stratified analyses for the association between TUG1 expression with overall survival (OS) (**A**) Subgroup analysis of HRs of OS by system of cancer. (**B**) Subgroup analysis of HRs of OS by factor of residence region.

Afterwards the stratified analyses were performed by factor of cancer type and residence region to analyze the possible sources of the heterogeneity (Table 2). For studies evaluating OS in different type of cancer, the results suggested that promoted lncRNA TUG1 levels could estimate worse outcome in bladder cancer (HR = 2.98, 95% CI: 1.84–4.83, *P* < 0.0001) and other system cancer (HR = 2.63, 95% CI: 1.42–4.87, *P* = 0.002), but not respiratory system cancer (HR = 0.93, 95% CI: 0.30–2.82, *P* = 0.895), (Figure 3A). Furthermore, we found a significant unfavorable association between elevated lncRNA TUG1 and OS of cancer patients in China (HR = 1.96, 95% CI: 1.12–3.44, *P* = 0.018) (Figure 3B). Significant heterogeneity was detected in the subgroups of cancer type (respiratory system and other system cancer) and residence region (China).

**Table 2:** Subgroup analysis of the pooled HRs of overall survival with over-expressed TUG1 in patients with cancer

Subgroup analysis	No. of studies	No.of patients	Pooled HR (95% CI)		Heterogeneity	
			Fix/Random	*p* Value	I^2^(%)	*p* Value
Cancer type						
Bladder cancer	2	101	2.98 (1.84,4.83)	< 0.0001	0.0%	0.973
Respiratory system	2	225	0.93 (0.30,2.82)	0.895	92%	0.000
Other system	4	514	2.63 (1.42,4.87)	0.002	72.9%	0.011
Residence region						
China	7	793	1.96 (1.12,3.44)	0.018	86.5%	0.000

**Figure 4:**
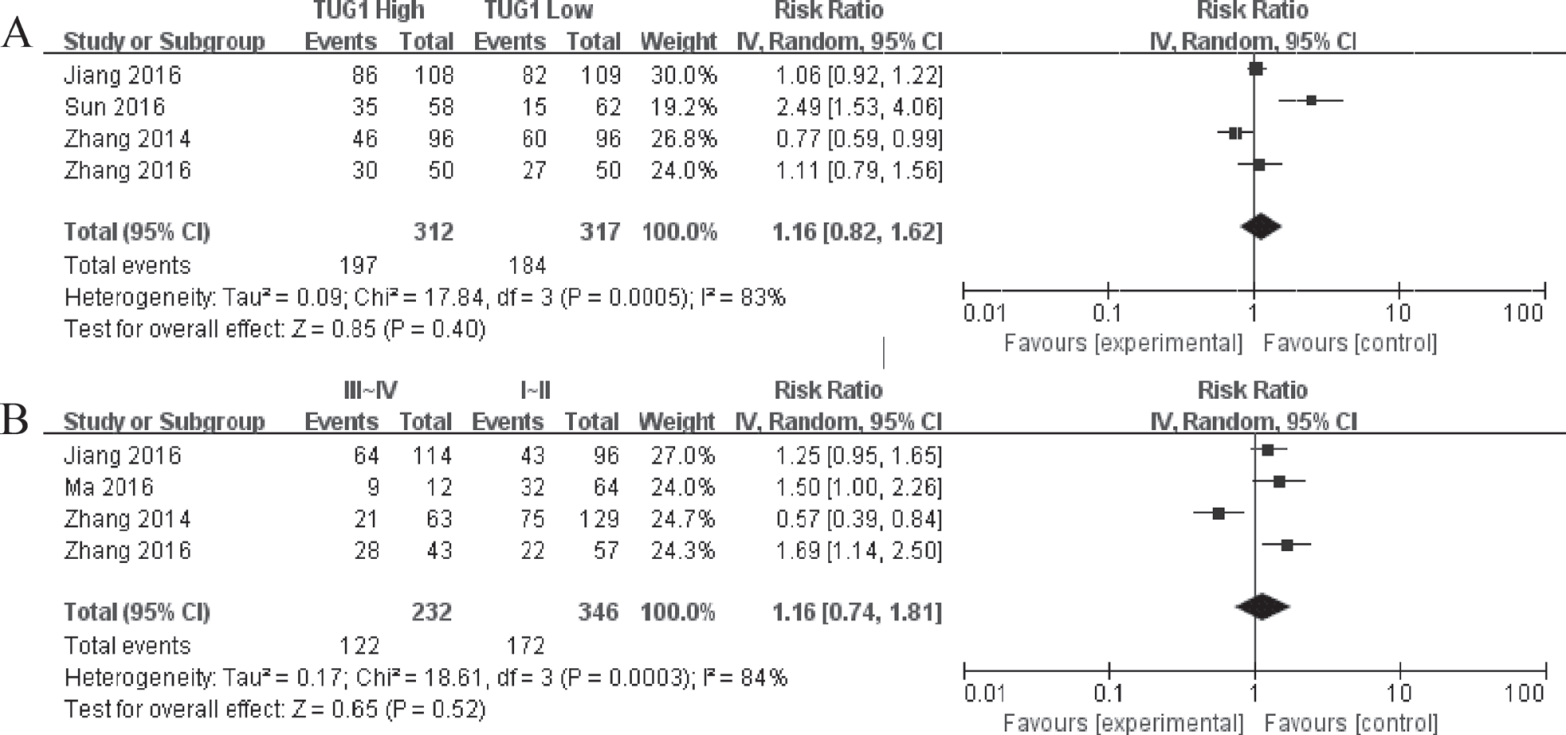
Forest plot for the association between TUG1 expression with lymph node metastasis (**A**) and TNM stage (III/IV vs. I/II (**B**)).

Subsequently, we set out to throw light upon the prognostic role of lncRNA TUG1 with lymph node metastasis and tumor progression in various cancers. The characteristics of the involved studies which evaluating the association between lncRNA TUG1 levels with tumor progression and lymph node metastasis were summarized in Figure 4. Through comparing the incidence of lymph node metastasis and tumor progression between high and low lncRNA TUG1 expression group by random model, we found that patients with increased lncRNA TUG1 levels failed to show incline to lymph node metastasis (HR: 1.16, 95% CI: 0.82–1.62, *P* = 0.40) and tumor progression (III/IV vs. I/II: HR 1.16, 95% CI: 0.74–1.81, *P* = 0.52).

### Publication bias

**Figure 5:**
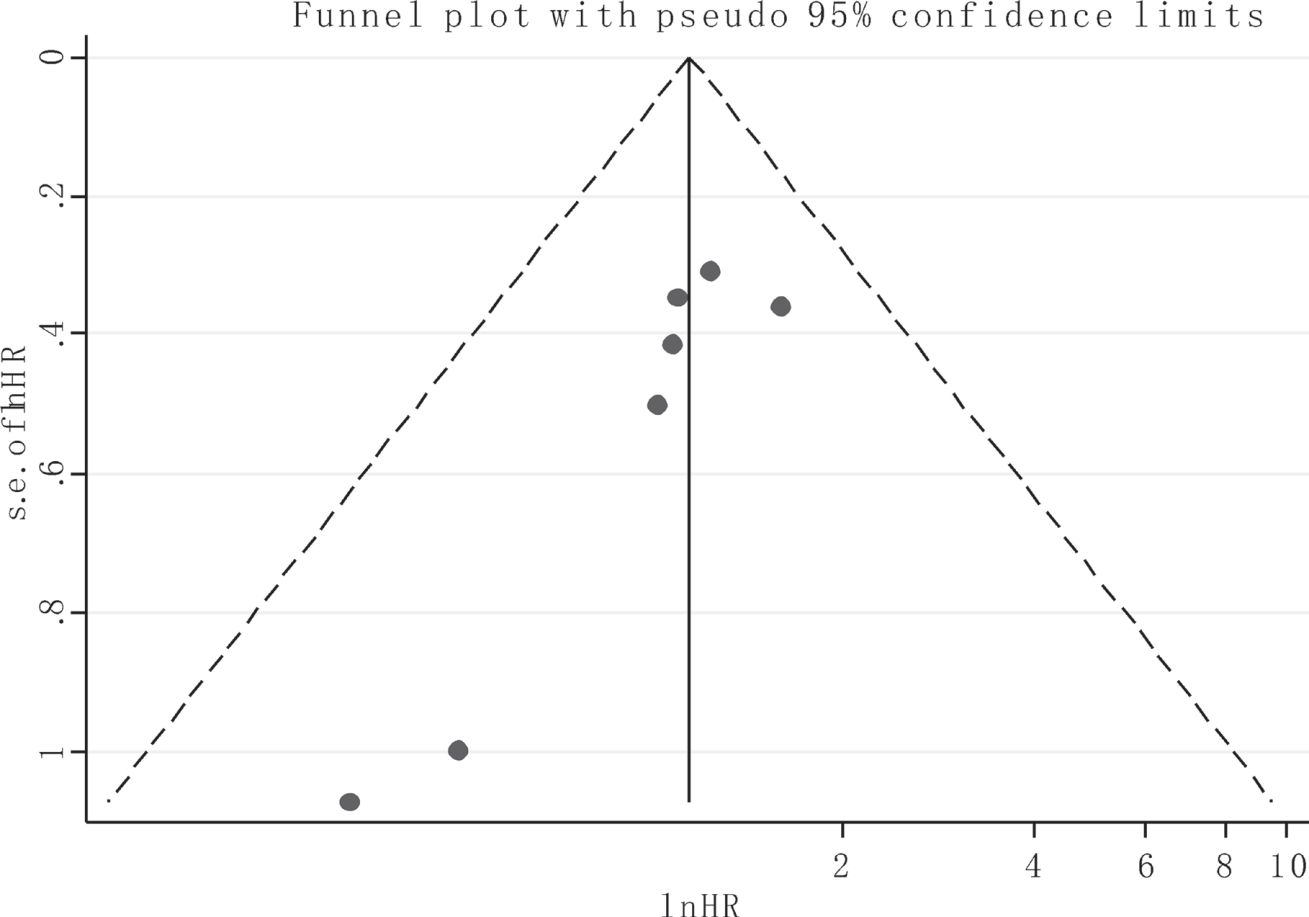
Funnel plot of the publication bias for overall survival

Publication bias of the included analyses was assessed by funnel plot and Begg's bias test. As expected, the shape of the funnel plot was symmetrical and the *P* value of the Begg's test was 0.108 for OS of all enrolled articles, suggesting the absence of significant publication bias in the meta analysis (Figure 5).

### Sensitivity analysis

**Figure 6:**
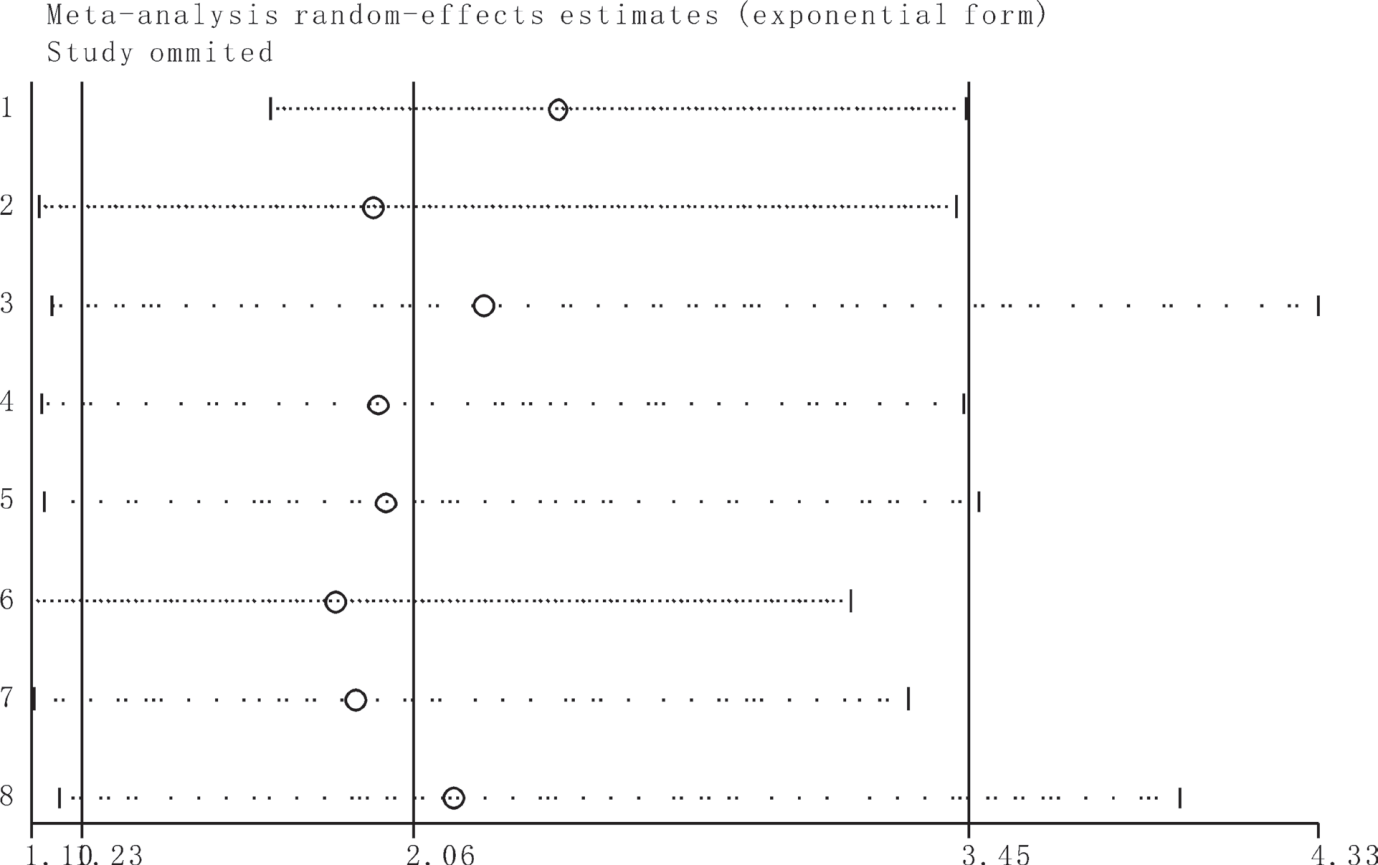
Sensitivity analyses of studies concerning TUG1 and overall survival

Sensitivity analysis indicated that the conclusions are stable because the pooled HRs was not significantly affected by the exclusion of any single study (Figure 6).

## DISCUSSION

Recently, as the functions of tumor-associated lncRNAs have gradually been characterized, it was demonstrated that lncRNAs may participate in the tumorigenesis and disease progression [21, 22]. Moreover, accumulating evidences have suggested that the dysregulation of cancer-specific lncRNAs could serve as novel prognostic biomarkers to more precisely evaluate the prognosis of different tumors [23, 24]. One of these hot lncRNAs is cancer-associated lncRNA TUG1.

LncRNA TUG1 is commonly expressed in human tissues and cancer cells. The mechanism underlying the relationship between elevated lncRNA TUG1 expression and poor prognosis in cancer patients need to be further elucidated. Recent studies manifested that lncRNA TUG1 contribute to the tumor progression through its regulation of diverse cellular processes, including migration, invasion, proliferation, differentiation, and apoptosis [25]. As a tumor suppressor in glioma, lncRNA TUG1 could activate caspase-3 and caspase-9 dependent pathways and suppress Bcl-2 dependent anti-apoptosis pathway [26]. In line with these findings, silencing of lncRNA TUG1 significantly upregulates the expression of the apoptosis-inducing factors such as AIF, AIP1, NIP3, and NIPOR, and suppresses cell growth and promotes apoptosis [15]. Therefore, lncRNA TUG1 was considered as anti-apoptotic factor similar to the role of survivin [27]. Additionally, lncRNA TUG1 has been demonstrated to function as a competing endogenous RNA (ceRNA) that binds to and reduces the expression of a number of miRNAs. The reduction of miR-9-5p, miR-144, miR-145 and miR-229 leads to tumor progression through the miR-9-5p/POU2F1, miR-144/c-Met, miR-145/ZEB2 and miR-229/VEGFA signaling pathway [14, 28–30].

These studies consistently suggested that overexpression of lncRNA TUG1 was a convinced unfavorable prognosis factor in small cell lung cancer, esophageal squamous cell carcinoma, bladder Cancer, gastric cancer, colorectal cancer and osteosarcoma. Conversely, Zhang *et al*. reported that lncRNA TUG1 expression was down-regulated in non-small cell lung cancer tissue and correlated with poorer OS. Since inconsistent evidence existed about the association of lncRNA TUG1 with OS, the current comprehensive meta analysis was performed to examine the clinical prognostic role of lncRNA TUG1 in a variety of carcinomas. A total of 8 studies including 840 patients were included in this study, and the results suggested that promoted lncRNA TUG1 expression was moderately correlated with poor prognosis in patients with various types of cancer. The analysis showed a pooled HR was 2.06 (95%CI: 1.23–3.45, *P* = 0.006), 1.16 (95%CI: 0.82–1.62, *P* = 0.40) and 1.16 (95% CI: 0.74–1.81, *P* = 0.52) for OS, LNM and TNM stage (III/IV vs. I/II), respectively.

Nonetheless the result of the current study should be interpreted with caution because there are several limitations should be considered. First, statistical heterogeneity was detected in the current study. The heterogeneity was probably due to the differences in the cancer type, the clinical characteristics of patients (country, age, tumor stage, etc.), the cut off value of lncRNA TUG1, the sample size, the time of follow-up, and so on. Of note, the prognostic value of lncNA TUG1 in lung cancer was inconsistent and need to be further elucidated. For example, Zhang *et al*. demonstrated that lncRNA TUG1 was down-regulated in non small cell lung cancer compared with the corresponding adjacent normal tissue and low lncRNA TUG1 expression in NSCLC was associated with a poor prognosis [13]. In line with this finding, Lin *et al*. found that knockdown of lncRNA TUG1 significantly promoted the proliferation of NSCLC cells maybe through in-trans regulation of Elav-like family member 1 (CELF1) [31]. Conversely, Niu *et al*. exhibited that lncRNA TUG1 was overexpressed in small cell lung cancer, and its expression was correlated with clinical stage and shorter survival time of SCLC patients [20]. The divergence was probably ascribed to remarkably disease specific expression pattern of lncRNA TUG1 than protein-coding genes [13]. Second, some of the HRs was calculated by reconstructing survival curves to extract the HR estimates rather than directly obtained from the primary studies. Finally, priority must be given to the cut off definition of lncRNA TUG1 expression among different investigations, which is the principal issue need to be resolved before its clinical application.

In conclusion, despite the limitations described above, it is preliminarily concluded that elevated lncRNA TUG1 is significantly associated with OS in cancer patients, and may be considered as a potentially and promising unfavorable prognostic factor in human cancers. Nevertheless, well designed large sample studies with specific cut off value will be necessary to verify and strengthen the prognostic role of lncRNA TUG1 in cancer patients.

## MATERIALS AND METHODS

### Search strategy and literature selection

Articles up to February 20, 2017, which related to the lncRNA TUG1 as a potential eligible biomarker for the prognosis of cancer patients, were comprehensive searched in several electronic databases, including Cochrane Library, PubMed, Embase, BioMed Central, Springer, ScienceDirect, ISI Web of Knowledge, together with three Chinese databases: China National Knowledge Internet (CNKI), Weipu and Wanfang. Publications with the following keywords were included: (“long noncoding RNA- ” OR “lnc RNA-” OR “noncoding RNA-” OR “taurine upregulated gene 1” OR “TUG1”) AND (“cancer” OR “carcinoma” OR “tumor” OR “neoplasm”) AND (“prognosis” or “prognostic” or “survival” or “metastasis”). The reference lists of primary publications were manually viewed to obtain additional relevant articles.

### Inclusion and exclusion criteria

Inclusion criteria: 1) Definite diagnosis or histopathology confirmed for patients with cancer; 2) Articles investigating the expression pattern of lncCRNA TUG1 in any malignant tumor; 3) Sufficient information for the computation of hazard ratios (HR) and corresponding 95% confidence intervals (CI).

Exclusion criteria: 1) Basic research; 2) Studies of non dichotomous lncRNA TUG1 expression or absence of survival outcome; 3) Multiple duplicate articles about a study, excluding earlier and smaller sample data; 4) Animal experiments, case reports, correspondences, editorials, expert opinions, letters, review articles and talks without original data.

### Data extraction and quality assessment

Two authors (XC and JL) reviewed each eligible article and extracted the data independently. All of the differences and contradictions were resolved by a third investigator. The major information from each enrolled study was extracted: (1) last name of first author, publication year, study design, country, cancer type, total cases, stage, follow-up time; (2) lncRNA TUG1 assessment method and specimen resources; (3) hazard ratio (HR) with 95% confidence interval (CI) of lncRNA TUG1 for overall survival, patient number for TNM state and progression, lymph node metastasis or distant metastasis. If univariate and multivariate analysis were both provided by the eligible studies, multivariate analysis was preferred because multivariate values have higher precision on interpreting confounding factors. If the data were only provided as Kaplan–Meier survival curves, the survival rates were extracted from the graphical plots and the estimations of HRs and 95% CI were then determined as the previously described method [32, 33]. MOOSE checklist was used to evaluate the quality of included publications [34] (Supplementary Table 1).

### Statistical analysis

The impact of lncRNA TUG1 expression on overall survival, lymph node metastasis, TNM stage and progression was examined by HRs and 95% CIs. An observed HR > 1 indicated poorer prognosis in patients with elevated lncRNA TUG1 expression and should be statistically significant when the 95% CI did not overlap with 1. The random-effects model was conducted to analyze the relationship between lncRNA TUG1 expression and clinical outcomes when calculated *I*^2^ > 50% [35–37]. Probable publication bias was examined by a funnel plot or conducting Begg's bias test [38]. *P* values < 0.05 was considered statistically significant. All statistical analyses were performed using Stata SE 12.0 (Stata Corporation) and RevMan 5.3 software.

## SUPPLEMENTARY TABLE




