# Supplementary material for: Prognostic role of lncRNA TUG1 for cancer outcome: Evidence from 840 cancer patients
Source: Oncotarget. 2017 May 13;8(30):50051–60. doi: 10.18632/oncotarget.17844 (PMC5564827; doi:10.18632/oncotarget.17844)
Supplement: Supplementary file 1 [file oncotarget-08-50051-s001.pdf]

Prognostic role of lncRNA TUG1 for cancer outcome: Evidence from 840 cancer patients

Supplementary Materials

Supplementary Table 1: MOOSE checklist. See Supplementary_Table_1
